# Comparison of step-count outcomes across seven different activity trackers: a free-living experiment with young and older adults

**DOI:** 10.1186/s13102-024-00943-0

**Published:** 2024-07-18

**Authors:** Takashi Nakagata, Yosuke Yamada, Masashi Taniguchi, Hinako Nanri, Misaka Kimura, Motohiko Miyachi, Rei Ono

**Affiliations:** 1 Department of Physical Activity Research, Health and Nutrition, National Institutes of Biomedical Innovation, Kento Innovation Park, NK Building, 3-17 Senrioka Shinmachi, Settsu-city, 566-0002 Osaka Japan; 2 Laboratory of Gut Microbiome for Health, Microbial Research Center for Health and Medicine, Health and Nutrition, National Institutes of Biomedical Innovation, 7-6-8, Saito-Asagi, Ibaraki City, 567-0085 Osaka Japan; 3 Institute for Active Health, Kyoto University of Advanced Science, 1-1 Nanjo Otani, Sogabe-cho, Kameoka- city, Kyoto, 621-8555 Japan; 4 Human Health Sciences, Graduate School of Medicine, Kyoto University, 53-Kawahara-cho, Shogoin, Sakyo- ku, Kyoto, 606-8507 Japan; 5 Department of Nursing, Doshisha Women’s College of Liberal Arts, 97-1 Minamihokotate, Kodo, Kyotanabe- city, Kyoto, 610-0395 Japan; 6 Faculty of Sport Sciences, Waseda University, 2-579-15 Mikajima, Tokorozawa-city, 359-1192 Saitama Japan

**Keywords:** Accelerometer, Assessment, Pedometer, Physical activity, Surveillance

## Abstract

**Background:**

There are now many different types of activity trackers, including pedometers and accelerometers, to estimate step counts per day. Previous research has extensively examined step-count measurements using activity trackers across various settings while simultaneously wearing different devices.; however, older adults frequently display distinct walking patterns and gait speeds compared to younger adults. This study aimed to compare the step-count between older and younger adults by having them simultaneously wear seven different activity trackers in free-living experiments.

**Methods:**

This study included 35 younger adults (21–43 yrs) and 57 physically independent older adults (65–91 yrs). All participants simultaneously wore one pedometer and six activity trackers: ActiGraph GT3X + Wrist and Hip, Omron Active Style Pro HJA-350IT, Panasonic Actimarker, TANITA EZ-064, Yamasa TH-300, and Yamasa AS-200 for seven days. A regression equation was also used to assess inter-device compatibility.

**Results:**

When comparing wrist-worn ActiGraph to the six hip-worn activity trackers, the wrist-worn ActiGraph consistently recorded step counts over 4,000 steps higher than hip-worn activity trackers in both groups (range, 3000–5000 steps). Moreover, when comparing the ActiGraph worn on the wrist to that worn on the hip, the proportion was higher among older adults compared to younger ones (younger: 131%, older: 180%). The Actimarker recorded the highest average step counts among six hip-worn devices, with 8,569 ± 4,881 overall, 9,624 ± 5,177 for younger adults, and 7,890 ± 4,562 for older adults. The difference between the hip-worn ActiGraph and Active Style Pro was just about 70 steps/day overall. The correlation among all devices demonstrated a very high consistency, except for the wrist-worn ActiGraph (*r* = 0.874–0.978).

**Conclusions:**

Step counts recorded from seven selected consumer-based and research-grade activity trackers and one pedometer, except for the wrist-worn ActiGraph. showed a variation of approximately 1700 steps (range, 1265–2275 steps) steps for both groups, yet maintained a high correlation with each other. These findings will be valuable for researchers and clinicians as they compare step counts across different studies or representative surveys conducted globally.

**Supplementary Information:**

The online version contains supplementary material available at 10.1186/s13102-024-00943-0.

## Introduction

Objectively measured daily step counts are among the simplest indices of daily physical activity [1]. Currently, various types of activity trackers, including traditional hip-worn devices, smartphones, and wrist-worn devices, are available to evaluate physical activity. These devices are categorized based on where they are worn on the body and the internal mechanisms used to detect steps, such as a spring-suspended lever arm, an accelerometer, or a piezoelectric sensor. The mechanisms, accuracy, and sources of error for various types of step counters worn by individuals in diverse settings, including structured laboratories, real-world environments, and simulated free-living conditions (such as field tests and treadmill exercises with or without controlled gait speed have been evaluated using video recording and hand-tally counts [2–5]. Additionally, recent studies have examined the accuracy and validity of step counts [6–8].

In a study on the differences in step counts obtained from multiple activity trackers, we recently reported the outcomes of 13 selected consumer-based and research-grade activity trackers, both under structured conditions in a metabolic chamber and during 15-day free-living trials [9]. Step counts obtained from 13 selected consumer-based and research-grade wearable devices varied within approximately 2500 steps for both the standardized day and free-living condition trials, even when worn concurrently by the same individual. Moreover, the wrist-worn accelerometers counted significantly more steps than did the other devices in both trials. Nonetheless, their measurements maintained a high correlation in the free-living trials. The findings of our previous study, which included regression equations, will be valuable for researchers, health professionals, and clinicians involved in prescribing exercise programs, as they can convert the step counts obtained with one device to the step counts that would be obtained with the other devices.

Nonetheless, our previous study had limitations in terms of the sample size, with only 19 participants; additionally, the inclusion criteria were limited to normal-weight individuals aged between 21 and 50 years. Consequently, the generalizability of these findings to the general population is limited. Particularly in older adults, it is well known that walking patterns and gait speeds differ from those of younger adults [10–12]. Moreover, previous studies highlighted the underestimation of step counts in experimental settings involving slow gait speed trials [4, 5, 13].

This study had two primary objectives: (1) to explore the variance in step-count measurements among six hip-worn and one wrist-worn activity trackers in older adults relative to younger adults, and (2) to derive equations essential for converting step counts from one device to another, utilizing standardized major axis analysis.

## Methods

### Participants

This study included 35 young adults aged 21 to 43 years and 57 older adults aged 65 to 91 years who were independently living in the community and could walk unassisted (Table 1). The older participants were selected from individuals who took part in a physical fitness assessment in Kyoto, while the younger participants were drawn from current students, alumni, and staff of Kyoto University. Prior to the study, the participants provided written informed consent after receiving information about the procedures and purpose of the study. All the procedures were reviewed and approved by our institute (no. kenei-198 m). All the participants provided written informed consent.

**Table 1:** Characteristics of participants

Characteristic	Overall, *N* = 92^1^	Young, *N* = 35^1^	Older, *N* = 57^1^	*p*-value^2,3^
Gender				0.993
Female	50 (54%)	19 (54%)	31 (54%)	
Male	42 (46%)	16 (46%)	26 (46%)	
Age, yrs	59.2 (25.7)	27.6 (6.4)	78.5 (6.8)	< 0.001
Height, cm	160.0 (9.4)	165.0 (9.5)	156.9 (8.0)	< 0.001
Weight, kg	54.4 (9.0)	56.6 (9.1)	53.0 (8.8)	0.082
BMI, kg/m^2^	21.2 (2.5)	20.7 (1.9)	21.5 (2.8)	0.087
Fat mass, kg	12.5 (4.4)	12.3 (4.4)	12.7 (4.4)	0.627
%fat, %	23.0 (7.2)	21.8 (7.5)	23.7 (7.0)	0.245
Fat free mass, kg	41.9 (8.0)	44.3 (9.0)	40.4 (7.1)	0.036
Skeletal muscle mass, kg	39.6 (7.7)	41.9 (8.6)	38.2 (6.7)	0.038

^1^n (%); Mean (SD)

^2^Pearson’s Chi-squared test; Wilcoxon rank sum test

^3^Significantly different older versus younger participants

### Experimental design

Participants visited the laboratory in the morning after an overnight fast of at least 10 h, underwent height, weight, and body composition measurements, and were asked to live their normal lives for seven days. All participants were equipped with seven devices, including six activity trackers worn on their hip and one pedometer: ActiGraph GT3X+ (ActiGraph LLC, Penascola, FL, USA), Omron Active Style Pro HJA-350IT (OMRON HEALTHCARE, Kyoto, Japan), Panasonic Actimarker EW4800 (Panasonic, Osaka, Japan), TANITA EZ-064 (TANITA, Tokyo, Japan), and Yamasa TH-300 (Yamasa, Tokyo, Japan). Of these devices, ActiGraph, Omron Active Style Pro HJA-350IT, and Panasonic Actimarker EW4800 are research-grade wearable devices [14–16], while TANITA EZ-064 and Yamasa TH-300 are consumer-based devices. In addition, the participants wore a Yamasa pedometer (ALNESS 200 S AS-200, Yamasa Corp., Tokyo, Japan), which is based on the response of a spring-mass system to gravity. Furthermore, the Yamasa pedometer (internationally known as Yamax) has been used in the National Health and Nutrition Survey conducted by the Ministry of Health, Labour and Welfare in Japan since 1989 [17, 18]. Positioning was randomly chosen for each participant as appropriate to minimize possible bias owing to placement, and each participant placed the devices in the same position throughout the experiment. All activity trackers, except for the ActiGraph and Yamasa pedometer, were concealed with tape to prevent participants from viewing metrics like step counts.

All devices were initialized before the trial and downloaded according to the manufacturer’s specifications using software provided by the respective companies. Before the experiment, the participants’ biometric information (such as age, sex, height, and weight) was input, with the exception of the Yamasa AS-200 pedometer (owing to lack of memory function). Participants wore all the devices during their waking hours but were instructed not to wear them during water-related physical activities or other physical activities during which the devices were difficult to wear. All participants were instructed not to wear any of the devices during this time. Furthermore, participants documented non-wear periods in a diary (e.g., bathing, showering) and recorded daily step counts using the Yamasa pedometer, which lacked a memory function, each evening during the free-living experiments. This diary guided the exclusion of data from seven days due to: (1) atypical days (such as travel or illness), and (2) unrecorded personal data due to device malfunctions. For instance, if data from the Omron Active Style Pro HJA-350IT were absent for a day, we discarded the data from all devices for that day, irrespective of whether other devices had successfully recorded the data. In addition, individuals with fewer than 100 steps or over 50,000 steps were excluded according to the National Health and Nutrition Survey in Japan [18]. Data from the initial day of activity monitor distribution was excluded from analysis for all participants. Ultimately, we analyzed 3073 datasets from 3864 datasets (92 participants, 6 days, and 7 devices).

### Anthropometry and body composition

Height and body weight were measured on the experimental days. The heights of the participants were then objectively measured to the closest 0.1 cm using an analog height meter. Body weight was measured, and appendicular lean mass was estimated using multifrequency bioelectrical impedance analysis (MF-BIA) (MC-780 A-N, TANITA, Tokyo, Japan), which was then validated using dual-energy X-ray absorptiometry [19]. Participants were evaluated in their underwear and were asked to stand barefoot on toe-and-heel electrodes while holding handgrips, with their arms hanging a few centimeters from the hips. The body mass index was calculated as weight/height^2^ (kg/m^2^).

### Statistical analyses

Statistical analyses were conducted, and figures were generated utilizing RStudio software for Mac (R version 4.2.2 [2022-10-31 ucrt], RStudio, Inc.). This statistical process proceeded in two sequential steps as follows.

(1) Comparison analysis: To describe the distribution of step counts across all devices, box plots were illustrated overall and by age groups. For descriptive statistics, values of continuous variables are expressed as mean and standard deviation (SD) or median and interquartile range (IQR). One-way analysis of variance (ANOVA) and post-hoc multiple comparison tests were conducted to analyze significant differences among all devices. Given that step counts can be used as an overall index of how active a person is [1, 20], the step counts obtained from each activity tracker were categorized into the following four categories: <5000, 5000 to 7499, 7500 to 9999, and ≥ 10,000. Moreover, to assess the difference in step counts between wrist-worn and hip-worn ActiGraph devices, the differences were calculated by subtracting hip-worn ActiGraph values from wrist-worn ActiGraph values. Subsequently, an unpaired *t*-test was conducted for the young and older groups. To visualize the difference in step counts, the “raincloud plot” was used [21].

(2) Association analysis: to examine the relationship between the step-count estimates of all devices, Pearson correlation coefficients were used based on the data distributions of all devices using the “ggpairs” function of the GGally package. Standardized major axis analyses were conducted to derive equations for converting step counts obtained with one device to those obtained with other devices, instead of using linear regression analyses. Standardized major axis analysis is a statistical method that accounts for errors in both the x-axis (independent variable) and the y-axis (dependent variable), enabling the assessment of error components related to both accuracy and precision [22, 23].

The figures were produced using the R package “ggplot2.” The level of statistical significance was established at 0.05.

## Results

Table 1 shows the participants’ characteristics, including fat mass and skeletal muscle mass. Among all the participants in this study, approximately 60% and 40% were female and male, respectively, with a similar distribution observed among younger and older adults.

### Descriptive statistics

**Fig. 1:**
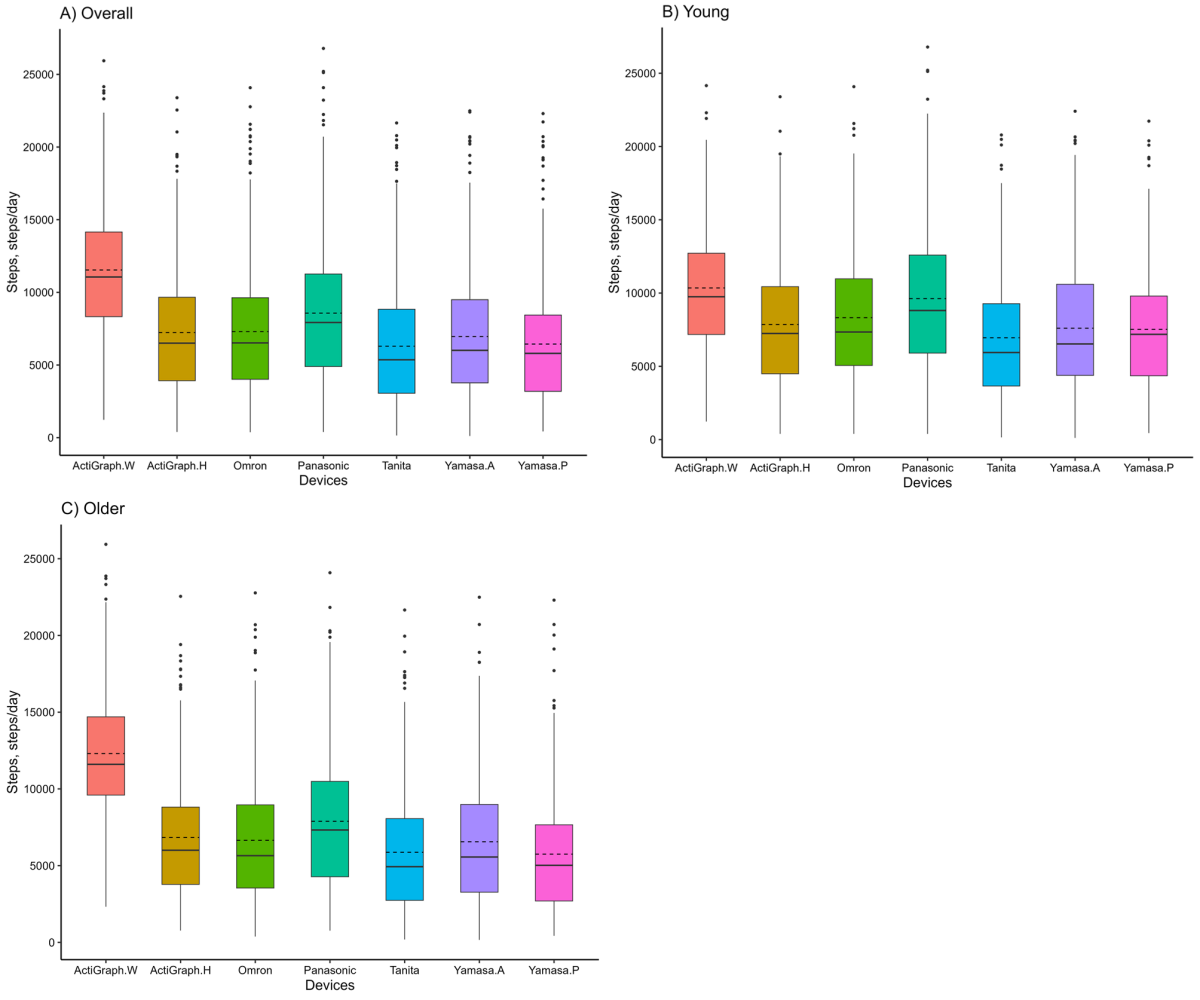
Box plots showing the daily number of steps completed during the 6-day free-living trial for seven activity trackers. Each box represents the interquartile range (IQR) of steps for the device, with the central notch indicating the median. Whiskers extend from the box to show the range of the data, excluding outliers. Dashed lines across the boxplots represent the mean number of steps recorded for each device

The step-count estimates of all devices in the free-living, including the median and quartile, are reported in Fig. 1; Table 2 and supplementary Table 1. The step count obtained by the ActiGraph worn on the wrist was the highest among the seven devices, and this result was consistent for both younger and older adults. Among six hip-worn devices, excluded ActiGraph wrist-worn, the Panasonic Actimarker EW4800 with a triaxial accelerometer was the highest value overall, registering 8,569 ± 4,881 steps/day (mean ± SD). Moreover, the Panasonic Actimarker EW4800 was highest in both age groups among six hip-worn, recording an average of 9,624 ± 5,177 steps/day in younger adults and 7,890 ± 4,562 steps/day in older adults. Conversely, the Yamasa AS-200 pedometer, which is based on the response of a spring-mass system to gravity, showed the lowest overall step count among the devices (6,442 ± 4,24 steps/day), ranking second to last in younger adults (7,517 ± 4,388 steps/day) and recording the lowest count in older adults (5,750 ± 4,015 steps/day). In the comparison between the hip-worn ActiGraph and the Omron Active Style Pro HJA-350IT, both of which are research-grade models, the average number of steps per day was roughly the same for both younger and older adults. Regarding TANITA EZ-064 and Yamasa TH-300, which are activity trackers of consumer-based, the number of steps obtained by Yamasa was higher than that of TANITA among the overall, younger, and older adults. With regard to the step count categories, the wrist-worn ActiGraph categorized approximately 60% into the category of 10,000 steps or more in overall (Supplementary Table 2).

**Table 2:** Daily number of steps completed during the 6-day free-living trial

Group	ActiGraph.W	ActiGraph.H	Omron	Panasonic	Tanita	Yamasa.A	Yamasa.*P*	*p*-value^#^
Overall	11,539 ± 4,334 [11,058, 8,331 − 14,155]	7,234 ± 4,291 [6,504, 3,923-9,662]	7,304 ± 4,562 [6,519, 4,020 − 9,631]	8,569 ± 4,881 [7,927, 4,900 − 11,263]	6,294 ± 4,327 [5,360, 3,057 − 8,836]	6,963 ± 4,440 [6,008, 3,774-9,496]	6,442 ± 4,249 [5,800, 3,189-8,439]	< 0.001
Young	10,347 ± 4,294 [9,747, 7,175 − 12,715]	7,854 ± 4,383 [7,242, 4,495 − 10,437]	8,319 ± 4,683 [7,342, 5,059 − 10,972]	9,624 ± 5,177 [8,810, 5,903 − 12,589]	6,949 ± 4,396 [5,944, 3,657-9,275]	7,598 ± 4,564 [6,527, 4,382 − 10,601]	7,517 ± 4,388 [7,182, 4,356-9,795]	< 0.001
Older	12,307 ± 4,192 [11,602, 9,598 − 14,697]	6,835 ± 4,191 [6,004, 3,778-8,815]	6,651 ± 4,368 [5,650, 3,542-8,961]	7,890 ± 4,562 [7,323, 4,277 − 10,489]	5,872 ± 4,237 [4,933, 2,738-8,066]	6,554 ± 4,317 [5,562, 3,272-8,987]	5,750 ± 4,015 [5,021, 2,698-7,662]	< 0.001

Mean ± standard deviation [Median, 25-75%]

^#^ Kruskal-Wallis rank sum test

**Table 3:** Standardized major-axis regression between the step-count estimations of all the devices, free-living trial

Model	Regression Equation
Yamasa.P ~ Yamasa.A	y = 0.85x + 501
Yamasa.P ~ Tanita	y = 0.86x + 1043
Yamasa.P ~ Panasonic	y = 0.77x − 181
Yamasa.P ~ Omron	y = 0.82x + 441
Yamasa.P ~ ActiGraph.H	y = 0.88x + 58
Yamasa.P ~ ActiGraph.W	y = 0.63x − 873
Yamasa.A ~ Tanita	y = 1.00x + 649
Yamasa.A ~ Panasonic	y = 0.86x − 440
Yamasa.A ~ Omron	y = 0.93x + 151
Yamasa.A ~ ActiGraph.H	y = 1.01x − 359
Yamasa.A ~ ActiGraph.W	y = 0.73x − 1469
Tanita ~ Panasonic	y = 0.81x − 668
Tanita ~ Omron	y = 0.90x − 251
Tanita ~ ActiGraph.H	y = 0.98x − 809
Tanita ~ ActiGraph.W	y = 0.68x − 1550
Panasonic ~ Omron	y = 1.00x + 1256
Panasonic ~ ActiGraph.H	y = 1.07x + 803
Panasonic ~ ActiGraph.W	y = 0.80x − 668
Omron ~ ActiGraph.H	y = 1.01x − 37
Omron ~ ActiGraph.W	y = 0.73x − 1171
ActiGraph.H ~ ActiGraph.W	y = 0.73x − 1196

**Fig. 2:**
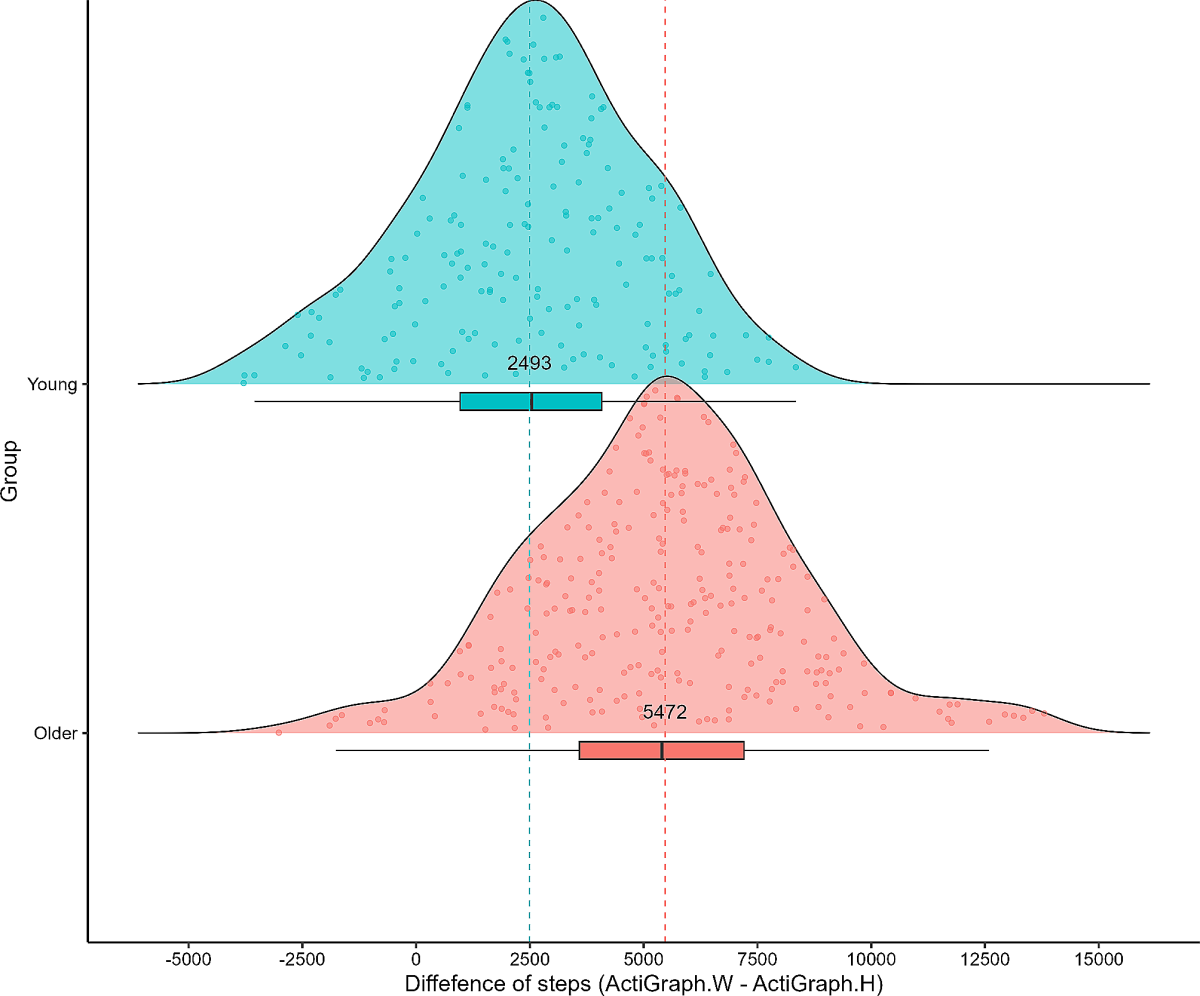
A raincloud plot illustrating the difference values between wrist-worn and hip-worn ActiGraph. This plot combines boxplot and ridgeline (density) plots. The centreline in the box represents the median, the lower hinge represents 25% quantile, and the upper hinge indicates 75% quantile. The dash lines represent the mean difference values of young and older, respectively

Our findings additionally reveal a more pronounced disparity in step counts between the hip and wrist among older adults compared to their younger adults. To be specific, our results demonstrate that wrist-worn ActiGraph devices record 131% more steps (an additional 2493 steps) than hip-worn ActiGraph in the case of younger adults (Fig. 2; Table 2). Conversely, for older adults, wrist-worn ActiGraph devices register a 180% increase (equivalent to 5472 additional steps) compared to hip-worn. The difference in ActiGraph step counts between the hip and wrist was significantly larger in older adults than in younger adults (*p* < 0.001).

### Correlations of all devices

**Fig. 3:**
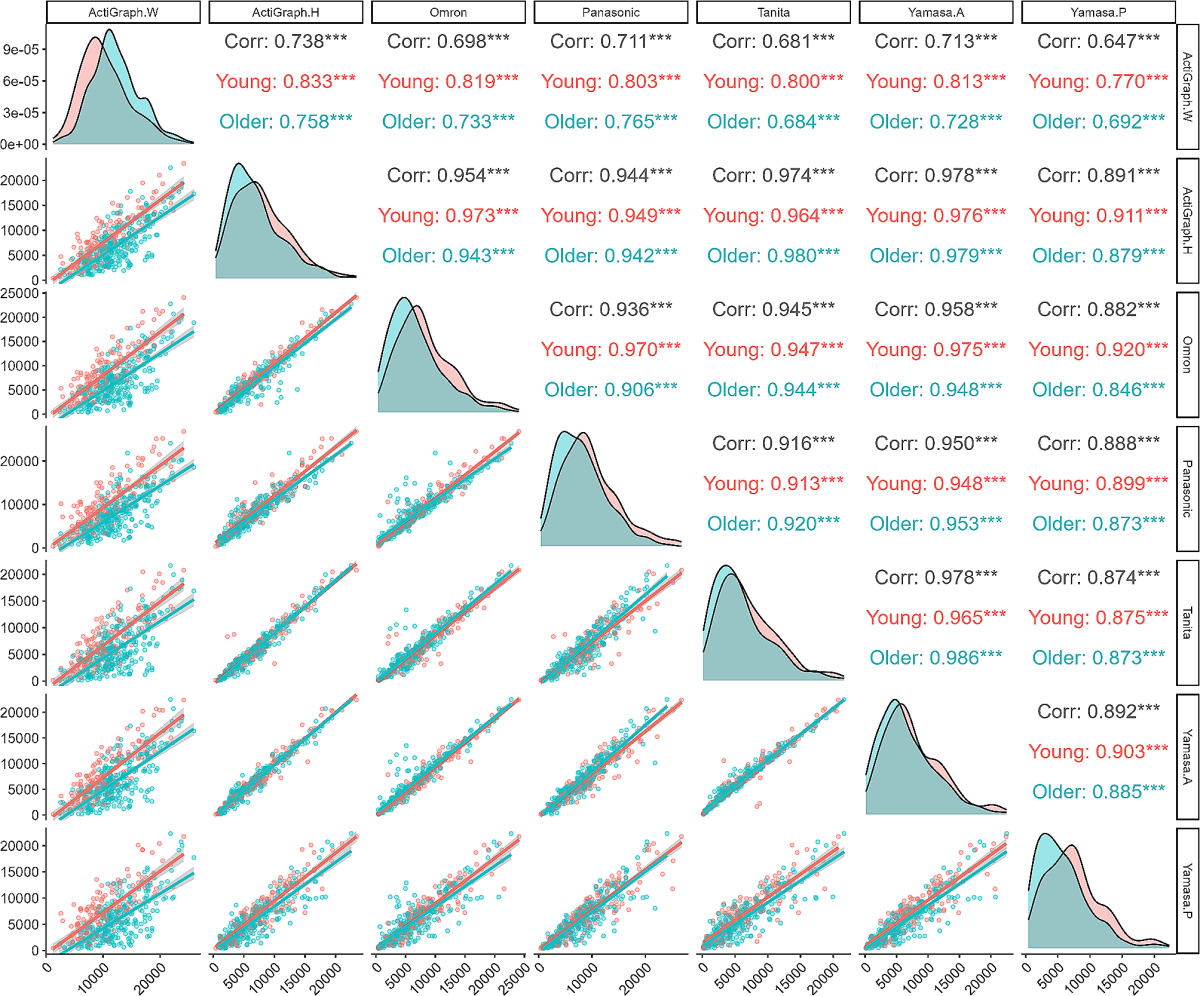
Paired scatterplot matrix of step-count estimates from all devices during free-living trials (lower section; scatter plots, diagonal section; distributions of each activity trackers, upper section; Pearson’s correlation coefficient and significant deference). In the upper section, the Gray color represents the correlation of Overall (Younger and Older), the red color means younger, and green means older adults. Corr, correlation coefficient. ***, Significant at *P* < 0.001

Figure 3 displays a paired scatterplot matrix created with the “ggpairs” function from the GGally package. It visualizes the correlation between step counts obtained from seven different devices, with the lower section showing scatter plots, the diagonal section showing distributions, and the upper section indicating correlations. In this scatterplot matrix, the slopes of the regression lines in the corresponding plots generally indicated a strong correlation, except for the wrist-worn ActiGraph (range of correlation in wrist-worn ActiGraph in overall; 0.647–0.738). The regression equations between the wrist-worn ActiGraph and hip-worn all devices differ between older and younger adults, however, the regression equations for the six hip-worn devices closely matched each other between younger and older adults, except for spring-mass pedometer (r range, 0.87–0.99, Fig. 1). Whereas, some values deviated significantly from the relationship in the case between the spring-mass pedometer and other devices (r range, 0.73–0.83).

### Standardized major-axis regression

The regression equations for converting the step counts obtained from all the devices using standardized major-axis regression are listed in Table 3.

## Discussion

The main findings of the present study were as follows: (1) Wrist-worn ActiGraph GT3X devices tended to record higher step counts than did hip-worn activity trackers in free-living experiments among both young and older adults (range, 3000–5000 steps), with low correlations observed between the other devices (r range, 0.647–0.738); and (2) the mean step counts from the six hip-worn devices showed a variation of approximately 1700 steps (range, 1265–2275 steps), but most of these devices showed strong correlations among both young and older adults.

To the best of our knowledge, this study is the first to explore the differences and associations in step counts across seven selected consumer and research-grade wearables during 6-day free-living trials, involving both younger and older adults. However, the current study has a major limitation. We lacked criterion or gold standard measurements, like the actual number of steps determined through visual observation (e.g., video recording, hand-tally count), or the use of devices like StepWatch worn on the ankle [2, 8, 24]. Therefore, this study was unable to compare step counts with a criterion or gold standard measurements gold standard; instead, it solely presents the correlations among different devices. In the research-grade model in our study, the ActiGraph is one of the most widely used accelerometer brands worldwide, and the Omron Active Style Pro HJA-350IT is especially popular in Japan. However, we recognize a significant limitation in our study due to the lack of reference or gold standard measurements and emphasize the necessity for further research using these standards. We have discussed the differences and associations of step counts between devices here.

First, we will discuss the difference in step counts between wrist-worn and hip-worn activity trackers. Wrist-worn accelerometers are increasingly being used as an objective measure of physical activity in various research surveys, in addition to traditional hip-worn devices. However, previous studies have reported that the step counts of wrist-worn activities were higher than those of hip-worn activities [25, 26]. For example, Gall et al. conducted a meta-analysis and found that wrist-worn activity trackers recorded an average of 3,537 more steps per day than hip-worn trackers in both young and older adults, across a total of 19 studies [25]. In addition, Mandigout et al. demonstrated that wearing the ActiGraph GT3X on the wrist may lead to overestimated step counts compared with the same ActiGraph model placed on the hip in 22 young and 22 older adults (mean ± SD: age, 27.2 ± 6.2 years and 76.6 ± 4.7 years, respectively) in free-living conditions [27]. Furthermore, the mean difference in step counts was 4,337 steps overall (wrist-worn: 11,203 ± 4,543; hip-worn: 6,866 ± 4,655), with the difference between wrist-worn and hip-worn devices being larger in older adults than in younger ones [27]. Additionally, Toth et al. [28] conducted a systematic literature review in adults aged 18 years or older, revealing a difference in step counts between the hip and wrist, especially highlighting the distinction between older and younger adults. The study reports that, in comparison to younger adults (showing a 140% capture, equivalent to 2795 more steps with wrist-worn monitors than hip-worn monitors), older adults exhibit a more pronounced contrast with a 189% capture (translating to 4360 more steps with wrist-worn monitors than hip-worn monitors) [28]. This difference can likely be attributed to activities that require movement of hands and wrists with little or no movement of the legs or feet, such as eating, desk work, and folding laundry [29]. Our results are consistent with those of previous studies, indicating that wrist-worn ActiGraph devices consistently registered higher step counts than did hip-worn activity trackers in free-living experiments involving both young and older adults (Fig. 2). Additionally, it was observed that the difference in step counts between the wrist and hip was more pronounced among older adults compared to their younger counterparts, and the regression equations for younger and older adults differed. During NHANES 2003–2006, ActiGraph devices (AM-7164) worn on the hip were used to investigate physical activity, including step count [30]. However, in NHANES 2011–2014, this protocol was changed, and participants were directed to wear the ActiGraph GT3X + on their wrist [31]. Direct comparison is challenging as both the location and the models of the devices differ; however, our results may help adjust step counts from both hip- and wrist-worn ActiGraph devices through regression equations.

Second, our study demonstrated that the mean step counts from the six hip-worn devices showed a maximum variation of approximately 1700 steps (range, 1265–2275 steps). In our study, both older and younger participants recorded higher step counts using the Panasonic Actimarker compared to other devices, while the YAMASA pedometer and TANITA device consistently showed lower step counts. Furthermore, the difference between the hip-worn ActiGraph and Active Style Pro was just about 70 steps/day overall. These results are similar to our previous study [9], which showed that step count by Panasonic Actimarker was almost 1500 steps higher than YAMASA and TANITA in free-living trial. Regarding the variations in daily step counts between devices, the differences may be due to a random-movement filter function that only counts steps when continuous walking is detected [32]. Notably, regarding the filter function of the activity trackers, the Omron HJ-720ITC, for example, employs a criterion that counts minimal steps within a 4-second interval [33]. Furthermore, the consumer-oriented devices (TANITA and YAMASA) feature proprietary filters with similar functions. Specifically, the TANITA device uses a mechanism to mitigate measurement errors by distinguishing the onset of walking (descending from each company’s website). If sustained motion persists for a duration of 7 s or more, the device categorizes it as walking and promptly presents aggregated measurement values up to that point. Additionally, after the cessation of motion, fresh measurements were not logged unless another continuous motion event of 7 s or longer occurred. In contrast, with the Yamasa device, steps were not counted within the initial 10 steps from the commencement of walking to avoid registering vibrations caused by placing the device in a pocket, bag, or other non-walking vibrations. In the event of uninterrupted walking, the total step counts up to that moment was immediately displayed. Therefore, activities that do not involve continuous walking, such as brief movements covering only a few steps, wolud be excluded from step counting. The observed discrepancy could result in notable differences in step counts when compared to those obtained from other devices, as it might exclude the accumulation of certain activities. Nevertheless, a high correlation was noted among all hip-worn devices, as indicated by a correlation coefficient varying between approximately 0.88 and 0.98 for both older and younger groups (Fig. 2).

### Practical application

Steps are an essential unit of locomotion, and objectively tracking daily step counts represents one of the simplest and most direct methods to quantify daily physical activity [1]. There are currently many different types of activity trackers including smartphones and wrist-worn activity trackers to evaluate physical activity including step count over the 3 last decades [34]. However, our study emphasizes the importance of considering the specific types of activity monitoring devices used when comparing physical activity across studies, including step counts. Omron Active Style Pro HJA-350IT and Panasonic activity trackers, which are research-grade devices, have been widely used among many types of Japanese studies to evaluate physical activity, and sedentary behavior [15, 35–38]. Furthermore, NHNS in Japan continuously evaluated the step counts using a YAMASA pedometer from 1989 [18]. Contrastingly, ActiGraph stands out as a widely acknowledged activity monitor, employed not only in national surveillance programs like NHANES in the United States [31, 39, 40] and the Canadian Health Measures Survey in Canada [41] but also commanding significant attention globally. Although different devices have been used in national surveillance, there is a possibility of approximating conversions through regression equations. Standardized major-axis regression, as shown in Table 3, may assist in the comparison of step counts across various studies or representative surveys conducted in different countries using various activity trackers.

Second, given the consistent finding of higher step counts from wrist-worn devices, researchers, clinicians, and users of pedometers and accelerometers should consider the implications for interpreting physical activity levels, associations with health-related outcomes such as mortality, and potential bias in results due to misclassification. For example, in their systematic review and meta-analysis, Cavero-Redondo et al. focused on examining the relationship between daily step counts and arterial stiffness, as measured by pulse wave velocity (PWv), a reference standard. Their subgroup analysis by device type revealed a stronger association between daily steps and PWv in studies utilizing pedometers. Although they partly attributed these results to discrepancies in step estimation methods among different brands and models of accelerometers, they also suggested that differences in attachment sites could be a more plausible explanation [42]. Our findings, including standardized major axis analysis, could assist researchers and health professionals in interpreting how step counts from various activity trackers correlate with health outcomes. For example, based on step counts assessed at the wrist, there is a possibility of discrepancies where individuals classified as “Active” might be classified as " Somewhat active” when assessed at the waist. These findings may also inform the prescription of exercise programs and interventions aimed at achieving specific step-count targets to improve population health and physical activity levels. Given the emphasis on the types of activity monitors and attachment sites, additional cohort studies are required to investigate the relationship between health outcomes and variations in attachment sites.

However, our study has several limitations. First, the participants in our study were not randomly selected from the city, potentially introducing a selection as they may have been more health-conscious than the general population. Second, the study sample size was small, consisting of 35 young adults aged 21 to 43 years and 57 older adults aged 65 to 91 years, and restricted to individuals with a normal BMI (mean and SD: young adults 20.7 ± 1.9, older adults 21.5 ± 2.8), limiting the generalizability of our results to the general population. Furthermore, all the older adults in our study were living independently in the community and able to walk unassisted. Therefore, further research should focus on other populations, including individuals with obesity or limited mobility, as well as those residing in assisted living facilities, to generalize the findings of this study. Finally, we lacked structured laboratory data for all participants, such as treadmill walking at controlled speeds or standardized protocols in a metabolic chamber. Thus, future studies should compare step counts under standardized experimental conditions, ensuring consistency in physical activity details and time spent on each activity across all participants.

## Conclusion

Step counts obtained from seven selected consumer-based and research-grade wearable devices varied within approximately 1700 steps under free-living condition trials but were still highly correlated with each other, except for the ActiGraph wrist. These results will be useful to researchers and clinicians facilitating the comparison of step counts across various studies or representative surveys conducted in different countries.

### Electronic supplementary material

Below is the link to the electronic supplementary material.

Supplementary Material 1

Supplementary Material 2

## Data Availability

Data are available for research purposes upon reasonable request to the corresponding author.
