# Supplementary material for: Comparison of step-count outcomes across seven different activity trackers: a free-living experiment with young and older adults
Source: BMC Sports Sci Med Rehabil. 2024 Jul 18;16:156. doi: 10.1186/s13102-024-00943-0 (PMC11264768; doi:10.1186/s13102-024-00943-0)
Supplement: Supplementary file 2 — Supplementary Material 2 [file 13102_2024_943_MOESM2_ESM.docx]

Supplementary table 2. Step count categories based on the modified Tudor-Locke and Bassett^#^

| Group | Category^#^ | ActiGraph.W | ActiGraph.H | Omron | Panasonic | Tanita | Yamasa.A | Yamasa.P | p-value^2^ |
| --- | --- | --- | --- | --- | --- | --- | --- | --- | --- |
| Overall | <5000 | 20 (4.6%) | 164 (37%) | 152 (35%) | 113 (26%) | 204 (46%) | 169 (38%) | 184 (42%) | <0.001 |
|  | 5000 to 7499 | 59 (13%) | 99 (23%) | 110 (25%) | 87 (20%) | 98 (22%) | 107 (24%) | 102 (23%) |  |
|  | 7500 to 9999 | 92 (21%) | 72 (16%) | 75 (17%) | 96 (22%) | 53 (12%) | 58 (13%) | 75 (17%) |  |
|  | ≥10000 | **268 (61%)** | 104 (24%) | 102 (23%) | 143 (33%) | 84 (19%) | 105 (24%) | 78 (18%) |  |
| young | <5000 | 13 (7.6%) | 52 (30%) | 43 (25%) | 31 (18%) | 68 (40%) | 54 (31%) | 52 (30%) | <0.001 |
|  | 5000 to 7499 | 35 (20%) | 39 (23%) | 45 (26%) | 32 (19%) | 39 (23%) | 48 (28%) | 40 (23%) |  |
|  | 7500 to 9999 | 46 (27%) | 35 (20%) | 31 (18%) | 42 (24%) | 27 (16%) | 20 (12%) | 40 (23%) |  |
|  | ≥10000 | **78 (45%)** | 46 (27%) | 53 (31%) | 67 (39%) | 38 (22%) | 50 (29%) | 40 (23%) |  |
| Older | <5000 | 7 (2.6%) | 112 (42%) | 109 (41%) | 82 (31%) | 136 (51%) | 115 (43%) | 132 (49%) | <0.001 |
|  | 5000 to 7499 | 24 (9.0%) | 60 (22%) | 65 (24%) | 55 (21%) | 59 (22%) | 59 (22%) | 62 (23%) |  |
|  | 7500 to 9999 | 46 (17%) | 37 (14%) | 44 (16%) | 54 (20%) | 26 (9.7%) | 38 (14%) | 35 (13%) |  |
|  | ≥10000 | **190 (71%)** | 58 (22%) | 49 (18%) | 76 (28%) | 46 (17%) | 55 (21%) | 38 (14%) |  |

^1^n (%)

^2^Pearson's Chi-squared test

^#^ Tudor-Locke C etal. Int J Behav Nutr Phys Act, 2011, Bassett DR, Jr. et al. Sports Med, 2017
